# Housing and overdose: an opportunity for the scale-up of overdose prevention interventions?

**DOI:** 10.1186/s12954-017-0203-9

**Published:** 2017-12-06

**Authors:** Geoff Bardwell, Alexandra B. Collins, Ryan McNeil, Jade Boyd

**Affiliations:** 1 British Columbia Centre on Substance Use, St. Paul’s Hospital, 608-1081 Burrard Street, Vancouver, BC V6Z 1Y6 Canada; 2 Department of Medicine, University of British Columbia, St. Paul’s Hospital, 608-1081 Burrard Street, Vancouver, BC V6Z 1Y6 Canada; 3 Faculty of Health Sciences, Simon Fraser University, 8888 University Drive, Burnaby, BC V5A 1S6 Canada

**Keywords:** North America, Overdose crisis, Housing, Overdose prevention interventions, Supervised consumption sites

## Abstract

**Background:**

North America is currently experiencing an overdose epidemic due to a significant increase of fentanyl-adulterated opioids and related analogs. Multiple jurisdictions have declared a public health emergency given the increasing number of overdose deaths. In the province of British Columbia (BC) in Canada, people who use drugs and who are unstably housed are disproportionately affected by a rising overdose crisis, with close to 90% of overdose deaths occurring indoors. Despite this alarming number, overdose prevention and response interventions have yet to be widely implemented in a range of housing settings.

**Overdose prevention interventions:**

There are few examples of overdose prevention interventions in housing environments. In BC, for example, there are peer-led naloxone training and distribution programs targeted at some housing environments. There are also “supervised” spaces such as overdose prevention sites (similar to supervised consumption sites (SCS)) located in some housing environments; however, their coverage remains limited and the impacts of these programs are unclear due to the lack of evaluation work undertaken to date. A small number of SCS exist globally in housing environments (e.g., Germany), but like overdose prevention sites in BC, little is known about the design or effectiveness, as they remain under-evaluated.

**Conclusions:**

Implementing SCS and other overdose prevention interventions across a range of housing sites provides multiple opportunities to address overdose risk and drug-related harms for marginalized people who use drugs. Given the current overdose crisis rising across North America, and the growing evidence of the relationship between housing and overdose, the continued implementation and evaluation of novel overdose prevention interventions in housing environments should be a public health priority. A failure to do so will simply perpetuate what has proven to be a devastating epidemic of preventable death.

## Background

Drug overdose deaths remain among the most pressing public health challenges in North America, which is currently experiencing an overdose epidemic. The proliferation of fentanyl-adulterated opioids and related analogs have given rise to an unprecedented overdose crisis, with opioid-related overdoses now the leading cause of accidental death in North America [1]. The overdose crisis has been particularly severe in the province of British Columbia (BC) in Canada, leading to the provincial declaration of a public health emergency in April 2016. That year, BC experienced approximately 1000 drug-related overdose deaths, which constitutes an 80% increase over 2015 [2]. In 2017, BC is on target to exceed this number of overdose deaths, with an estimate of 31.3 deaths per 100,000 individuals [2]. The public health response to the overdose epidemic in BC has proven to be challenging, particularly given the proliferation of fentanyl-adulterated opioids and related analogs that are driving rising overdose mortality across Canada [3].

People who use drugs (PWUD) and who are unstably housed have been disproportionally affected by the overdose crisis in British Columbia, as elsewhere [4]. According to a recent provincial coroner’s report, 88.5% of BC’s overdose deaths are occurring indoors, with men who use alone being most affected [2]. Despite these statistics, and the well-described aspects of housing environments that shape overdose risk [5–7], overdose prevention and response interventions have yet to be widely implemented in a range of housing settings, including in those designed specifically for PWUD. Furthermore, research has largely focused on substance use and housing for people experiencing homelessness rather than for those who are housed [8–10]. However, a recent systematic review of Housing First literature found an absence of explicit mention and discussion of harm reduction interventions [11]. With the exception of managed alcohol programs [12–14], there remains a lack of published research on harm reduction interventions that target drug use in housing environments specifically.

## Overdose prevention interventions

In BC, a small number of overdose prevention interventions have begun to be implemented within housing environments that serve as models to inform overdose responses in other settings. These include housing-based overdose prevention sites, peer-led naloxone training and distribution, peer witness injection programs, and shared-using rooms. Drug user organizations (e.g., Vancouver Area Network of Drug Users, Downtown Eastside SRO Collaborative) and other community groups have also scaled-up naloxone training and distribution within housing environments to ensure that PWUD are properly equipped and trained to administer naloxone in the event of an overdose [15]. Further, a city-funded naloxone pilot project has also been implemented in 12 low-income private housing buildings with the highest overdose rates in Vancouver, BC’s Downtown Eastside neighborhood. In this project, tenants are hired in each building as peer workers to provide naloxone training and distribution to residents and guests in their buildings [16].

Additionally, peer witness injection programs were established in two temporary winter emergency shelters in Vancouver from December 2016 to March 2017. In each shelter, a designated room was set up with a table, chairs, and harm reduction supplies (e.g., sterile water, syringes, and cookers) for shelter residents to inject drugs. Peer staff were hired part-time and trained to monitor drug use onsite and respond in the event of an overdose. Finally, “shared-using” rooms have been implemented in select supportive housing sites in Vancouver to expand witnessed injection programming to tenants, particularly those who are socially isolated. Shared-using rooms are spaces separate from residents’ personal rooms where they can go to inject drugs. While these rooms vary between buildings in how they are monitored (e.g., cameras or staff) or designed (e.g., designated rooms or hallways), they are intended to provide a ‘supervised’ space for people to use drugs, as an alternative to using alone in their rooms where they may be at greater risk of dying from an overdose [17]. Although these interventions constitute a positive step towards reducing overdose deaths in housing environments, their coverage remains context-specific and limited, they are often not officially sanctioned, and the precise impacts of these programs are unclear due to the lack of evaluation work undertaken to date.

Moreover, overdose prevention sites (OPS) have been established in communities across BC and are similar to supervised consumption sites (SCS) in that PWUD are monitored by trained staff or volunteers to intervene in the event of an overdose. Unlike SCS, which require an exemption to operate legally under current federal law, OPS operate under a provincial ministerial order given the public health emergency [18]. As such, OPS tend to be simpler in design and operation, are more peer-driven, and offer no or fewer clinical services. Since the implementation of OPS in BC, activists across Canada have opened similar OPS, although the legality of such sites remains unclear [19]. In BC, OPS exist in housing environments such as supportive housing and homeless drop-in centers [20, 21], but they too have not been well described. While some evaluations are currently underway, findings have yet to be published.

SCS currently exist in a variety of settings, with over 90 located in eight countries [22]. SCS are health settings where people can consume pre-obtained drugs under the supervision of medically trained staff [23]. These services have been rigorously evaluated and have been shown to reduce adverse health-related outcomes, including morbidity and mortality associated with overdose [24, 25]. Despite the overwhelming scientific evidence supporting SCS, there continues to be social and structural barriers (e.g., drug-related stigma and anti-harm reduction policies) to the implementation of these life-saving public health interventions in communities around the globe. In settings across North America, a lack of support by policymakers and governments to fund and implement evidence-based harm reduction interventions continue to pose major challenges to opening SCS. However, circumventing such political barriers has proven feasible within the context of a public health emergency. For example, in Canada, a shift in government policies has made it significantly less onerous to open SCS, given the severity of the overdose crisis [26]. Consequently, there has been a rapid scaling up of SCS, with multiple sites having opened in Montréal, Toronto, Ottawa, Victoria, Surrey, Kelowna, Kamloops, and Vancouver. Furthermore, provincial ministerial orders in BC have aided in the implementation of OPS, including funding for these services, and yet, other regions in Canada continue to face political barriers. Despite these challenges, several other North American cities are also considering the implementation of SCS as a measure to respond to overdose and other harms associated with injection drug use (e.g., San Francisco and New York) [27].

Given the known effectiveness of SCS in reducing overdose morbidity and mortality, it is somewhat surprising that these services have yet to be widely implemented and evaluated in housing environments, especially given the disproportionate rate of fatal overdoses occurring in private housing environments [2]. There are a small number of SCS within housing environments worldwide. For example, in Frankfurt, Germany, the “Eastside” facility is a large rehabilitation center that also offers SCS for its approximately 100 residents. Additionally, Luxembourg City, Luxembourg, has the “Abrigado”––a low-threshold housing facility that offers SCS along with HIV testing, counseling, and primary care services [28]. However, little is known about the design or effectiveness of these SCS, as they remain under-evaluated.

## Conclusions

Despite SCS developments, there have been few interventions for PWUD who are unstably housed. Implementing SCS and other overdose prevention interventions across housing models provides multiple opportunities to address overdose risk and drug-related harms for marginalized PWUD. For example, incorporating SCS in emergency shelters or supportive housing would provide services beyond the capacity of part-time staff and volunteers. This integration could include having medically trained staff onsite to provide harm reduction supplies and education, supervise drug use, make referrals to health and social services, and respond to overdoses and other health issues that may arise. Additionally, the rapid gentrification of urban areas highlight the need for innovative programming (e.g., mobile SCS and mobile distribution of harm reduction supplies) that extend support to displaced and vulnerably housed PWUD. Complementing fixed overdose prevention services, including those within housing environments, with more flexible interventions may provide a more effective response to the various overdose risks faced by PWUD who are vulnerably housed.

Until we can achieve more large-scale legal and policy-based harm reduction goals (e.g., decriminalization), the scaling up of SCS and other novel overdose prevention interventions in housing environments represents an innovative public health opportunity deserving of immediate attention. Further efforts should now be focused on the ways in which such interventions can be embedded in a range of housing and shelter environments to adequately respond to the overdose epidemic. These services should also be rigorously evaluated, including an examination of their impacts on rates of using alone and fatal overdose, impacts on residents who do not use drugs, effects on specific at-risk populations, and their cost effectiveness. Given the current North American overdose crisis, and growing evidence of the relationship between housing and overdose, the continued implementation and evaluation of novel overdose prevention interventions in housing environments should be a public health priority. A failure to do so will simply perpetuate what has proven to be a devastating epidemic of preventable death.

## Abbreviations

BC: British Columbia
OPS: Overdose prevention sites
PWUD: People who use drugs
SCS: Supervised consumption sites
